# 2232. Comparison of Empiric Vancomycin Prescribing in Response to Gram Positive Cocci Alerts on Blood Cultures Between Primary and Overnight Physicians on General Medicine Services: A Retrospective Study

**DOI:** 10.1093/ofid/ofad500.1854

**Published:** 2023-11-27

**Authors:** Abdulaziz Almulhim, Heather L Cox, Amy Mathers, Stacy Park

**Affiliations:** University of Virginia Medical Center, Charlottesville, Virginia; University of Virginia Health, Charlottesville, Virginia; University of Virginia, Charlottesville, VA; University of Virginia Medical Center, Charlottesville, Virginia

## Abstract

**Background:**

Blood culture contamination is associated with unnecessary antibiotic use, increased length of stay, and increased costs. Assessing the likelihood of true bacteremia versus contamination requires consideration of clinical context and microbiologic data. This may be more difficult for covering physicians overnight, who typically cover more patients and may not know patients’ clinical history as well. We compared rates of vancomycin (VAN) initiation in response to microbiology alerts for gram-positive cocci (GPC) in blood cultures during primary team shifts versus overnight.

**Methods:**

We queried the University of Virginia Clinical Data Repository for blood culture results positive for GPC from 07/01/2021 to 12/30/2022 in inpatient general medicine patients. Cultures were stratified by time of the initial positive gram stain alert, which is called as a critical result to the team. All new positive blood cultures are run on a rapid molecular diagnostic (ePlex GenMark Diagnostics) with speciation results passively entered into the chart 2 hours after gram stain. Results were excluded if the patient was already on VAN or daptomycin at the time of gram stain or if the gram stain resulted during hours when the physician coverage (primary vs covering) was not consistent. The primary outcome was the rate of VAN initiation within 2 hours of gram stain with GPCs. Secondary outcomes were number of repeat blood cultures, length of stay (LOS), and incidence of acute kidney injury (AKI).

**Results:**

320 instances of positive blood cultures were reviewed. 141 were excluded. Of 179 included instances, there was no significant difference in VAN initiation by overnight physicians compared to primary teams (21% vs 26%; p=0.418). There was no significant difference in repeat cultures or incidence of AKI.

Comparison of primary and overnight physicians

**Figure:**
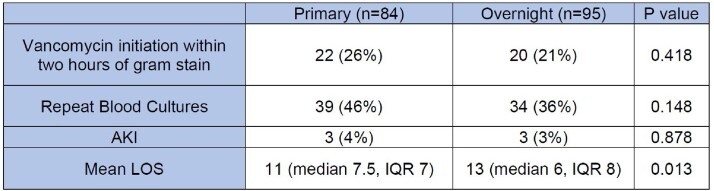

Blood culture speciation

**Figure:**
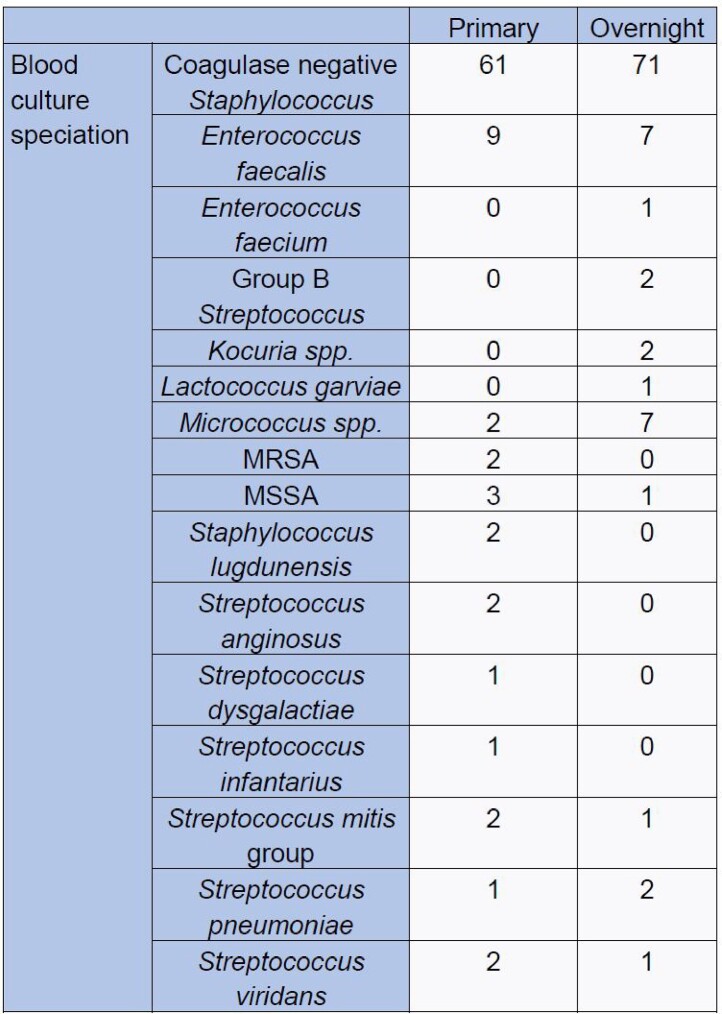

**Conclusion:**

There was no difference in VAN use, repeat blood cultures, or AKI when positive gram stain alerts were received by overnight physicians versus primary teams. Stewardship interventions targeting VAN prescribing in the setting of a rapid diagnostic workflow may not need to be specifically adapted to covering physicians or teams.

**Disclosures:**

**Amy Mathers, MD, D(ABMM)**, Merck: Advisor/Consultant

